# Effects of Low-Protein Diets and Exogenous Protease on Growth Performance, Carcass Traits, Intestinal Morphology, Cecal Volatile Fatty Acids and Serum Parameters in Broilers

**DOI:** 10.3390/ani9050226

**Published:** 2019-05-09

**Authors:** Gervais Ndazigaruye, Da-Hye Kim, Chang-Won Kang, Kyung-Rae Kang, Yong-Jin Joo, Sang-Rak Lee, Kyung-Woo Lee

**Affiliations:** 1 Department of Animal Science and Technology, Konkuk University, 120 Neungdong-ro, Gwangjin-gu, Seoul 05029, Korea; gervais@konkuk.ac.kr (G.N.); kdh142536@naver.com (D.-H.K.); leesr@konkuk.ac.kr (S.-R.L.); 2 SOLTON Biochem Inc., Seocho-gu, Seoul 06691, Korea; kkucwkang@konkuk.ac.kr (C.-W.K.); kkang@soltonbiochem.com (K.-R.K.); yongjjoo@soltonbiochem.com (Y.-J.J.)

**Keywords:** broiler chickens, exogenous protease, low-protein diet, growth performance

## Abstract

**Simple Summary:**

To maintain sustainability, the poultry production industry uses low-protein diets to minimize the potential for environmental pollution from nitrogen waste. However, reducing dietary crude protein may compromise the performance and health of the chickens. Thus, strategies using crystalline amino acids or exogenous protease added to the low-protein diets, the latter being the more recent trend, have been common practice in the industry, in order to meet the ever-increasing demand for poultry meat in the most sustainable way possible. In the present study, we evaluated a novel protease, produced by the bacterial species *Bacillus clausii* isolated from the body fluid of a polychaete (*Periserrula leucophryna*) found only in Korean waters, as an additive in broiler chickens fed the low-protein diets. We found that dietary protease improved growth performance in these broiler chickens.

**Abstract:**

Dietary exogenous proteases (ENZ) can be used in poultry production to improve the growth of chickens fed low-protein (LP) diets. We hypothesized that ENZ supplemented in an LP diet would improve growth performance and physiological response in broilers for 8–35 days. To investigate this, we used a 2 × 2 factorial design with crude protein (CP, normal diet (NP) and LP) and ENZ. The LP diet contained low in 1% CP and ca. 8–12% amino acids compared to the NP diet and both NP and LP diets were added without or with (1 g/kg of diet) ENZ. We randomly allocated 720 1-week-old Ross 308 male chicks to 48 pens and experimental diets. At 21 days, dietary ENZ, but not CP, increased (*p* = 0.007) live body weight. Body weight gain from 8–21 days was affected (*p* = 0.006) by dietary ENZ, but was not affected (*p* = 0.210) by CP. The feed conversion ratio was affected by both CP and ENZ during the starter period (*p* < 0.05), by ENZ (*p* = 0.034) during the finisher period, and by CP (*p* < 0.001) during the whole period. However, the interaction between CP and ENZ did not significantly affect growth performance (*p* > 0.05). Dietary ENZ increased (*p* = 0.013) the relative weight of liver at 21 days. CP and ENZ affected (*p* = 0.043) total short-chain fatty acids at 21 days. However, this effect was not seen (*p* = 0.888) at 35 days. Dietary CP increased (*p* < 0.05) the serum concentrations of both uric acid and creatinine in broilers. We concluded that dietary ENZ is more beneficial to younger broilers, independent of CP levels, and that its effect was restricted to body weight and the feed conversion ratio.

## 1. Introduction

Protein is the second most substantial nutrient and the most expensive component of poultry diets. Protein in modern poultry production practice is predominantly sourced from soybean meal (SBM), which is high in protein with well-balanced amino acids coupled with high digestibility. This meal meets approximately 80% of the protein and amino acid requirements of all types of poultry and swine in all life stages. Poultry needs a specific quantity and balance of essential amino acids (EAA) and nitrogen (for synthesis non-essential amino acids, NEAA) rather than crude protein [1]. Chickens fed the standard levels of dietary protein can synthesize NEAA from excess EAA. However, when low-protein (LP) diets are used, less EAA is available for NEAA synthesis. 

In monogastric animals, the digestion of protein is driven mainly by endogenous proteases through two stages of gastric digestion in an acidic environment, and pancreatic digestion in the small intestine. Although endogenous proteases synthesized and released in the gastrointestinal tract are usually sufficient to optimize feed protein utilization [2,3], a considerable amount of protein (18–20%) passes through the gastrointestinal tract incompletely digested [4,5,6,7]. Therefore, exogenous proteases (ENZ) have been introduced into livestock feed to improve its nutritive value [8].

Several ENZ are commercially available and their use has significantly increased in corn-SBM-based diets to improve growth performance of broiler chickens through improved protein and amino acid digestibility. Dietary ENZ can facilitate the utilization of proteins that are otherwise unavailable to the animal, especially when feed ingredients are low quality and/or have low bioavailability [9]. It has been reported that dietary ENZ enhances weight gain and feed efficiency by improving amino acid content and energy utilization and reducing proteolytic fermentation, bacterial toxins, and the amount of nutrients excreted in feces, thereby reducing potential environmental pollutants [10,11,12]. 

Additionally, proteases may cleave proteinaceous anti-nutrients, including trypsin inhibitors, thus leading to enhanced utilization and bioavailability of amino acids [10,13]. Although the beneficial effects of ENZ on chicken growth performance may be a result of the increased protein digestibility and capacity for energy utilization, they may also be a result of changes to gut morphological characteristics [14], or other gut health indicators, such as volatile fatty acids (VFA) and secretory immunoglobulin A or characteristics of the tibia or edible meat. However, to our knowledge, these aspects have not been previously studied. The objective of the present study was to assess the effects of ENZ supplementation of LP diets on growth performance, carcass yield, meat quality, ileal morphology, cecal volatile fatty acids, and serum parameters of broiler chickens. We measured a wide range of biological parameters in addition to production performance because Cowieson and Roos [8] suggested that dietary ENZ may have extra-proteinaceous effects in broiler chickens. In addition, given the fact that the broiler chicks grow rapidly, it is important to have two sampling intervals [15]. 

## 2. Materials and Methods 

The experimental procedures used in this study were approved by the Institutional Animal Care and Use Committee (IACUC) of Konkuk University (KUB182061).

### 2.1. Exogenous Protease

The ENZ (Pearlzyme^®^, PEARLZYME Co., Inchon-si, South Korea) used in this study was an extracellular alkaline protease produced by the bacterial species *Bacillus clausii* isolated from the body fluid of a Korean polychaeta (*Periserrula leucophryna*) living in the tidal mud flats of Gwangwha Island in the Korean West Sea (Yellow Sea). The ENZ has an optimum pH of around 9, but it can maintain its stability over a broad range from pH 5.5–12 and up to 50 °C [16]. The ENZ preparation (1 g ENZ per kg of diet) was added into the basal diets to exceed 10,000 U/kg of diet. The optimal inclusion level was previously evaluated in laying hens and broiler chickens [17,18]. 

### 2.2. Birds, Experimental Design, and Diets

A total of 850 1-day-old Ross 308 male broiler chicks were purchased from a local hatchery, housed into floor pens, and fed a commercial pre-starter mash diet for 7 days. From 8–35 days, chicks (*n* = 720/experiment) were assigned to one of four treatments with 12 replicates of 15 birds per pen. Care was taken to ensure an equal initial body weight among and within treatments. Isocaloric diets with two CP levels were formulated to have normal (NP) and LP (1% CP and ca. 8–12% amino acids) diets. In addition, both NP and LP diets were administered with or without ENZ. The experiment had 2 × 2 factorial arrangement of treatments with diets containing two levels of CP and two levels of ENZ supplementation. The experimental diets were in crumble form during the starter period and pellet form during the finisher period (Table 1), and contained carbohydrase, phytase, anticoccidials, and alternatives to antibiotics. The analyzed and calculated nutrient composition of the diets are presented in Table 1. Feed and water were available ad libitum. The room temperature was set at 33 °C during the first week of the trial and gradually decreased by 3 °C every week until 24 °C, at which it was maintained for the remainder of the experiment. The pens were kept under a 23:1 h (L:D) photoperiod. 

### 2.3. Sampling

Body weight and feed intake were measured weekly on a pen basis and was used to calculate the feed conversion ratio. Mortality was monitored daily and was used to correct the feed intake. At 21 days and 35 days, one bird from each pen close to the mean body weight was selected and euthanized by carbon dioxide (<15% of the chamber volume added per minute) for sampling blood. Serum samples were obtained by centrifugation (HA-1000, Hanil Industry, Korea) at 1700× *g* for 15 min and stored at −20 °C until use. Immediately after blood sampling, the left breast and leg meats, liver, pancreas, and spleen were also sampled, weighed, and expressed as relative organ weight to the live body weight. A segment of ileum (from Meckel’s diverticulum to the ileocecal junction) was sampled for morphological examination and measurement of secretory immunoglobulin A (sIgA) content. Finally, paired caeca were aseptically collected and stored on ice until the preparation for the VFA analysis. 

### 2.4. Measurement of Meat Quality

The breast and leg meats were evaluated for pH, meat color, and cooking loss 24 h postmortem. The pH was measured using a pH meter (Testo 205, Testo AG, Lenzkirch, Germany) in three different sites of the pectoralis major and thigh muscles.

The color of raw breast and leg meat samples was determined in the center of the muscle at three different points using a portable spectrophotometer (CM-2600d, Konica Minolta, Ramsey, NJ, USA). The International Commission on Illumination (CIE) lightness (L*), redness (a*), and yellowness (b*) components were obtained from the Specular Component Excluded (SCE) mode readings. To measure the cooking loss, the flesh of the left breast and deboned leg meat were placed into individual vacuum sealed plastic bags and cooked in a water bath at 80 °C for 30 min to reach an internal temperature of 70 °C, as described by Kim et al. [15]. After cooking, meat samples were cooled in ice-cold water for 10 min and residual moisture was removed with a paper towel before re-weighing. Cooking loss was considered to be difference between the weight of the uncooked and cooked sampled meats [19].

### 2.5. Tibia Characteristics 

The left tibia was prepared by manually removing the attached meat to reveal the tibia bone and weighing it. The diameter and length of the tibia were measured using a digital caliper. Tibia breaking strength was measured using an Instron universal testing machine (Model 3342, Instron Corp., Norwood, MA, USA) with a 50-kg load range and a crosshead speed of 50 mm/min with the tibia supported on a 3.35-cm span. The graph showed the plateau curve of applied maximal force (KN) to measure the bone strength as expressed as energy stored in the bone.

### 2.6. Gut Morphology

Intestinal segment samples (approximately 1 cm long) of ileum were excised, flushed with 10% neutral-buffered formalin to remove the contents, and fixed in 10% neutral-buffered formalin for a minimum of 48 h. The 4-μm sections were prepared and dyed with standard hematoxylin-eosin solution. The structure of the mucosa was observed at 40× magnification using an Olympus BX43 microscope (Olympus, Tokyo, Japan) and photographed using a digital camera (Olympus eXcope T500). At least 15 intact well-oriented crypts and villi were counted per section and used to calculate the villus height/crypt depth (VH/CD) ratio. 

### 2.7. Cecal Volatile Fatty Acid Analysis

Approximately 1 g of cecal digesta was added to 9 mL of cold distilled water and homogenized using a digital ultra-turrax T25 (IKA, Staufen, Germany). Added to the mixture were 0.05 mL of saturated HgCl_2_, 1 mL of 25% H_3_PO_4_, and 0.2 mL of 2% pivalic acid and centrifuged at 3000× *g* at 4 °C for 20 min. Then, the supernatant (1 mL) was collected and stored at −20 °C before analysis. The concentration of VFA in cecal samples was measured using gas chromatography (6890 Series GC System, HP, Palo Alto, CA, USA) as described previously [15]. 

### 2.8. Cecal Clostridium perfringens Counts

Approximately 1 g of cecal digesta was added to 9 mL of cold distilled water and was subjected to a 10-fold serial dilution. The dilutions were then spiral-plated on reinforced clostridial agar (Reinforced clostridial medium; BD Difco) and incubated anaerobically at 37 °C for 24 h. The number of characteristic black colonies was then counted and expressed as log_10_ cfu/g of cecal digesta. 

### 2.9. Ileal Secretory Immunoglobulin A (sIgA) Measurement

The ileal segment was longitudinally excised and rinsed using phosphate buffered saline (PBS) solution to remove digesta. The ileal mucosa was collected by gently scraping the gut mucosa surface using a tissue culture scraper and homogenized with 5 mL PBS, and then the mixture was centrifuged at 27,000× *g* at 4 °C for 20 min. The supernatants were then aliquoted and frozen at −20 °C until use. Total sIgA content was measured using a commercially available IgA ELISA kit following the manufacturer’s instructions and was expressed per cm of the intestine. 

### 2.10. Serum Biochemical Parameters

Serum samples were analyzed using an automatic blood chemical analyzer (Film DRI CHEM 7000i, Fuji film, Tokyo, Japan) for total cholesterol (TCHO), high-density lipoprotein (HDL) cholesterol, triglyceride (TG), total protein (TP), albumin (ALB), globulin (GLB), albumin: globulin (ALB:GLB), glutamic oxalacetic transaminase (GOT), glutamic pyruvic transaminase (GPT), uric acid (UA), and creatinine. In addition to the biochemical parameters, nitric oxide (NO) was also measured in the serum samples as described previously [20].

### 2.11. Statistical Analysis

Each replicate pen was considered to be an experimental unit. Data for all variables were analyzed by two-way analysis of variance (ANOVA) with the model including CP, ENZ, and their interaction as the main variables, using the PROC MIXED procedure of the SAS software (SAS 9.4, SAS institute). A random effect was not considered in the procedure. A multiple comparison test was used to compare among each treatment using the PDIFF option. Significance was considered at *p* < 0.05 and trends were noted when 0.05 < *p* < 0.10. 

## 3. Results

### 3.1. Growth Performance

The dietary ENZ significantly increased (*p* = 0.007) live body weight in broiler chickens at 21 days, but not at 35 days (Table 2). Dietary CP did not affect live body weight at any age. No significant effect of the interaction between CP and ENZ on live body weight was observed. Dietary ENZ significantly increased body weight gain from 8–21 days (*p* = 0.006). However, similar to the live body weight, dietary CP did not affect body weight gain at any age. Feed intake at all ages was not affected by either CP or ENZ. Both CP and ENZ affected the feed conversion ratio at 21 days (*p* < 0.05). LP vs. NP significantly increased the feed conversion ratio (*p* = 0.009), while dietary ENZ significantly decreased it at 21 days (*p* < 0.001). From 22–35 days, the feed conversion ratio was increased by dietary ENZ (*p* = 0.034), but not CP (*p* = 0.062). However, LP vs. NP significantly increased (*p* < 0.001) the feed conversion ratio during the whole period. No significant effect of the interaction between CP and ENZ on production parameters was observed. 

### 3.2. Relative Organ Weights

Both CP and ENZ did not affect spleen weight at 21 and 35 days (Table 3). At 21 days, dietary ENZ significantly increased the relative liver weight (*p* = 0.013); however, this ENZ-mediated increase in liver weight was not observed at 35 days. Dietary CP did not affect the liver weight at any age. Both CP and ENZ did not affect relative pancreas weight at either 21 days or 35 days. However, a significant effect of the interaction between CP and ENZ was observed at 35 days (*p* = 0.010): dietary ENZ increased the relative pancreas weight in the NP diet but had the opposite effect in the LP diet. 

### 3.3. Meat Yield and Quality

Leg meat yield was not affected by CP but decreased with ENZ at 21 days (Table 4). Leg meat yield decreased when ENZ was added to the LP diet leading to a significant interaction between CP and ENZ (*p* = 0.026). Cooking loss was not affected by either LP or ENZ. Dietary ENZ in the NP diet increased CIE L* value (*p* < 0.05) but decreased it in the LP diet (*p* < 0.05), thus causing the interaction between the two variables at 21 days (*p* = 0.030). At 35 days, dietary ENZ lowered CIE L* value (*p* < 0.05). Dietary ENZ significantly lowered CIE b* value at 21 days (*p* = 0.002). The pH of the leg meat was not affected (*p* > 0.05) by either CP or ENZ. 

Breast meat yield was not affected by CP or ENZ on any of the days (Table 5). A significant effect of the interaction between the two variables on cooking loss (*p* = 0.040), CIE L* (*p* = 0.021), and CIE b* (*p* = 0.049) was observed at 21 days. LP vs. NP increased CIE L* value at 21 days (*p* = 0.012). CIE a* value was not affected by CP and ENZ at any age. LP vs. NP decreased the pH of the breast meat at 21 days (*p* = 0.018), while dietary ENZ decreased it at 35 days (*p* = 0.017). 

### 3.4. Tibia Characteristics

Tibia characteristics, i.e., weight, diameter, and length, and breaking strength were not affected by CP or ENZ (Table 6), even though ENZ tended to decrease tibia weight at 35 days (*p* = 0.061).

### 3.5. Ileal sIgA Content, Morphometric Indices and C. perfringens Counts

Both CP and ENZ did not affect villus height, crypt depth, or the VH/CD ratio (Table 7). In addition, both CP and ENZ did not affect sIgA content on any day (Table 7). Although not significant, there was an effect of the ENZ and CP interaction on the sIgA content (*p* = 0.084). None of the variables tested affected the cecal population of *C. perfringens* (Table 7).

### 3.6. Volatile Fatty Acid Content in Cecal Digesta 

Both CP and ENZ did not affect cecal VFA at 21 days (Table 8). However, dietary ENZ in the NP diet substantially increased total short-chain fatty acids compared with those in the LP diet, thus causing the interaction (*p* = 0.043) between two variables. Although not statistically significant, marginal effects of the interaction between CP and ENZ were observed on acetate (*p* = 0.067), butyrate (*p* = 0.051), and valerate (*p* = 0.072). At 35 days, VFAs were not affected by CP or ENZ. 

### 3.7. Serum Biochemical Parameters

Dietary CP and ENZ did not affect most serum biochemical markers, i.e., TCHO, TG, HDL, LDL, TP, ALB, GLB, ALB: GLB, GOT, and GPT (Table 9). The LP diet significantly increased UA levels compared with those of the NP diet. Dietary ENZ marginally increased UA levels (*p* = 0.054). However, no effect of the interaction between the two variables on UA levels was observed. The LP vs. NP diet significantly increased the concentration of creatinine in serum samples (*p* = 0.028). NO levels in serum samples were not affected by either CP or ENZ (Table 9). 

## 4. Discussion

### 4.1. Growth Performance

Dietary CP levels did not affect live body weight, daily weight gain, or feed intake at any age. However, feed conversion ratios at 21 days and from 8–35 days were significantly impaired in chickens fed an LP diet compared with those on an NP diet. The latter finding was expected because the LP vs. NP diet increased body fat accumulation and/or decreased the efficiency of feed utilization [21]. Our findings partially agree with those of a previous study that reported low-CP diets negatively affecting the feed conversion ratio in broiler chickens [22]. 

In contrast to our expectation, there was no effect of the interaction between dietary ENZ and CP on performance traits. Rather, the beneficial effects of ENZ on live body weight, body weight gain, and feed conversion ratio observed at 21 days were independent of CP level. ENZ-mediated improvement of the feed conversion ratio at 21 days reversed slightly but significantly during the finisher period. The results of our study agree with those of previous studies [12,23,24] that reported the beneficial effects of ENZ on live growth performance during the growing phase of chickens. However, other studies have reported results that are in contrast to our study, and they reported a lack of ENZ effects on growth performance in broiler chickens [11,25,26]. 

In our study, dietary ENZ did not affect feed intake, but improved body weight and the feed conversion ratio, especially at 21 days. The enhanced body weight may in part be the consequence of the ENZ-mediated increase in nutrient digestibility. Although we did not measure this in our study, it has been commonly reported that dietary ENZ increased nutrient digestibility in broiler chickens [12,14,23,24,27,28,29]. One study reported that dietary ENZ improved the digestibility of CP and amino acids in broilers that had been fed maize-SBM-based diets [30]. Our results clearly show that the ENZ used in this study was stable during feed processing (e.g., pelleting), and that it contributed to the host-derived enzyme digestion process. Based on the lack of interactions between ENZ and CP, further studies are warranted to evaluate nutrient digestibility or changes in production traits caused by dietary ENZ in low-quality protein ingredients.

### 4.2. Relative Organ Weights

Dietary ENZ increased the relative weight of the liver at 21 days, and this is likely to be the consequence of the increased influx of absorbed nutrients leading to increased nutrient synthesis, although we did not measure the synthetic activities in hepatocytes. This finding is consistent with that of a recent study by Rehman et al. [31] who reported higher liver weights in birds that had been fed diets containing different sources of protease. Similarly, Erdaw et al. [32] revealed that adding microbial protease significantly increased the relative liver and pancreas weights at day 24 in broiler chickens. Conversely, other studies have reported no clear effect of dietary ENZ on the relative liver weight [33,34].

It has been reported that dietary exogenous enzymes added singly or in combination, decreased the pancreas weight in broiler chickens [35,36,37,38,39]. This indicates potentially important, but not yet fully understood, associations between in-feed exogenous enzymes and the pancreas. In contrast, we did not observe an ENZ effect on pancreas weight at 21 days. However, the significant interaction observed between ENZ and CP did affect the relative pancreas weight at 35 days. This interaction was caused to occur because the ENZ-addition effect on pancreas weight was either positive or negative depending on the level of CP. Owing to the crossover-type interaction, no overall effect by either variable was observed, and the observed means cannot be separated by pair-wise comparisons. The reasons for the effect of ENZ on pancreas weight depending on the CP level are not yet well understood and require further research. 

### 4.3. Meat Yield and Qualities 

Dietary ENZ in the NP diet increased cooking loss of breast meat at 21 days, leading to a significant interaction between ENZ and CP. It is not clear from our results how dietary ENZ negatively affected the cooking loss. In contrast to our study, Xu et al. [29] reported an increase in breast muscle weight and pH value and a decrease in breast muscle drip loss with protease supplementation in broiler chickens. A significant effect of the interaction between ENZ and CP on breast meat CIE L* and CIE b* values was noted at 21 days, and the pair-wise comparison revealed that paler breast meat (high CIE L* value) was produced in chickens fed with the LP diet in the absence of ENZ. These findings agree with those of Salakova et al. [40] who reported a negative correlation between pH and CIE L* values. For the CIE b* value, a crossover interaction was detected that drove the directional ENZ effect depending on the CP level. An interaction between ENZ and CP on the leg meat yield and the CIE L* value was observed at 21 days. Dietary ENZ did not affect leg meat yield in the NP diet but lowered it in the LP diet. The CIE L* value at 21 days exhibited a crossover interaction by factors: dietary ENZ increased the CIE L* value in the NP diet but lowered it in the LP diet. At 35 days, dietary ENZ significantly lowered CIE L* values at 35 days. Also, dietary ENZ significantly increased the CIE a* values at 35 days but decreased the CIE b* values at 21 days.

Many factors, including nutrition (e.g., CP, amino acids, fats, and pigments), affect the meat quality of broilers [41]. In the present study, the only independent variables used were CP and ENZ. Therefore, whether changes in the quality of breast and leg meats are reflected by ENZ-mediated increases in absorbed nutrients requires further investigation. However, we emphasize that for dependent variables (i.e., CIE L* for leg meat; cooking loss, CIE L*, and a* for breast meat) exhibiting crossover interactions, dietary ENZ in the LP diet improved meat quality (e.g., darker breast meat or lower cooking loss), while ENZ in the NP diet slightly impaired those traits. Thus, we speculate that the positive ENZ effects on meat quality in chickens fed the LP diet are partially mediated by increased absorbed nutrients. Further studies analyzing meat composition would shed a light on this speculation. 

### 4.4. Ileal Morphology and sIgA Content in the Ileum

Villus height is closely associated with nutrition absorption [42]. The crypt is considered the be the villi factory, and deep crypts indicate rapid tissue turnover and a high demand for new tissue [43]. It has been reported that dietary exogenous enzymes improved the gut morphology of broiler chickens [27,39,44]. However, the results of our study contradict these reports because we found that dietary ENZ did not affect the gut morphological indices that we measured at 21 days.

sIgA is the most abundant immunoglobulin in the small intestine and plays an important role in avian gut immunity [45,46]. In our study, sIgA was not altered by ENZ and CP at 21 days, but ENZ decreased sIgA content in chickens fed the LP diet, causing a moderate but non-significant interaction (*p* = 0.084) between ENZ and CP. Peng et al. (2016) postulated that increased sIgA enhances gut health, which may lead to increased nutrient absorption and improved growth performance [47]. However, increased sIgA content in gut digesta also indicates a response to inflammatory stimuli at the local tissues. Thus, in the LP diet, a low sIgA content coupled with ENZ may indicate an improved gut environment, which would decrease the demand for sIgA, and thus its production. In any event, neither CP nor ENZ influenced the gut morphology or sIgA content. However, it should be noted that all treated diets contained exogenous enzymes (i.e., carbohydrase and phytase), anticoccidials, and alternatives to antibiotics, which may decrease the effect of CP or ENZ on the gut parameters including ileal morphology measured. 

### 4.5. C. perfringens Counts and Short Chain Fatty Acids in Cecal Digesta

Short-chain fatty acids (SCFA), such as acetate, butyrate, propionate, and lactate [48,49], which are principal VFAs in the chicken gut, are produced during carbohydrate fermentation [47], while branched-chain fatty acids are a product of protein fermentation [50] in the gastrointestinal tract. Thus, we initially expected that either CP or ENZ might affect SCFAs in the cecal digesta because it would increase or decrease substrates for cecal microbes. However, our results were in contrast to our expectation, and we found no main factor effect; however, a significant interaction between ENZ and CP on total SCFA was observed. Owing to the presence of a crossover interaction, there was no difference detected between means, and the effect of ENZ on SCFAs was either increase or decrease depending on the CP level. However, a clear explanation is not forthcoming, and further research is required to reveal the details. 

We expected that ENZ supplementation would affect *C. perfringens* population in ceca by decreasing the levels of substrates in the distal gut. However, the results of our study revealed that cecal *C. perfringens* populations were not affected by ENZ or CP. These findings do not agree with a previous study by Yan et al. [51] who reported that dietary protease in combination with carbohydrase lowered ileal *C. perfringens* concentrations of 15-day-old broilers. Similarly, Kamel et al. [27] found that dietary protein levels and protease inclusion significantly decreased the total concentration of ileal *Clostridium* spp. in broiler chickens. The discrepancies between our results and those of previous studies are not readily apparent, although there were differences in the experimental settings. For example, in our study, we counted *C. perfringens* at 35 days, while Yang et al. [51] did this much earlier at 15 days. Furthermore, the diets used in these previous studies [27,51] did not contain antibiotic additives, while all the diets in our study contained many additives, including carbohydrase, phytase, anticoccidials, and alternatives to antibiotics. The presence of these additives may disguise any effects of ENZ on the parameters measured, and this should be further explored in future studies.

### 4.6. Blood Metabolite Profiles

Serum biochemical indices are often used to monitor health, and for the diagnosis and treatment of diseases, and also to reflect the nutritional status of chickens [52]. In our study, serum TG, ALB, TG, TCHO, HDL, GLB, GOT, and GPT were not affected by ENZ and CP. It was shown that, LP vs. NP increased UA and creatinine. Our findings are in sharp contrast to those of several previous studies [24,53,54,55,56], which reported a significant decline in UA in broiler chickens that were fed low-CP diets. In addition, dietary ENZ tended to increase UA, especially when added to the LP diet. It is often reported that serum UA concentrations can be used as an indicator of amino acid utilization in broilers fed amino acid-sufficient and amino acid-deficient diets [57]. Thus, a clear explanation on the increased UA as a result of the LP vs. NP diet is not forthcoming.

The LP vs. NP diet raised creatinine levels in serum samples of broilers. Creatinine is a product of creatine phosphate in muscle tissue, and its production is proportional to muscle mass. In agreement with our findings, previous research has found that high vs. low CP diet lowered serum creatinine levels in broiler chickens [58]. Dietary ENZ in both LP and NP diets marginally increased serum creatinine levels, but the effect was not significant. 

## 5. Conclusions

In our study, dietary ENZ improved growth performance, increased liver weight, and affected carcass traits in broilers. The ENZ-mediated increase in growth performance, independent of CP level, was clearly observed at 21 days. It is noteworthy that dietary ENZ affected meat quality either positively or negatively depending on the CP level. Dietary ENZ did not affect some parameters including ileal morphology, sIgA content, *C. perfringens* concentration, and serum biochemical indices. Our findings indicate that ENZ can be added to LP diets for broilers to enhance growth performance and increase production efficiency. Further studies are warranted in order to investigate whether ENZ alone or in combination with other enzymes is more effective at improving the performance, nutrient digestibility, and physiological response of broilers to diets containing low-quality protein ingredients. 

## Figures and Tables

**Table 1:** Ingredients and nutrient composition of the basal normal- (NP) and low-protein (LP) diets.

Ingredients, %	Starter		Finisher	
	NP	LP	NP	LP
Ground corn	47.73	49.51	48.49	48.84
Wheat, 10% crude protein (CP)	15.00	15.00	15.00	15.00
Soybean meal, 45.5% CP	22.50	22.57	26.12	26.19
Rapeseed meal	3.00	3.00	1.25	2.29
Corn gluten meal	3.00	1.33	-	-
Meat & bone, 40%	1.00	1.00	1.00	1.00
Poultry meal	2.00	2.00	2.00	0.04
Yellow grease	2.82	2.87	3.44	4.00
DL-methionine hydroxy analog	0.32	0.28	0.32	0.25
L-lysine sulfate, 70%	0.55	0.38	0.30	0.15
L-threonine, 98%	0.07	0.04	0.06	0.01
Salt	0.22	0.22	0.18	0.19
Limestone	0.91	0.91	0.90	0.95
Sodium bicarbonate	0.07	0.07	0.10	0.10
Vitamin and mineral premix ^1^	0.24	0.24	0.24	0.24
Liq. chol-cl (50%)	0.05	0.05	0.05	0.05
NSPase	0.05	0.05	0.05	0.05
Mono-dicalcium phosphate, 21%	0.40	0.41	0.43	0.58
Phytase	0.01	0.01	0.01	0.01
Alternative to antibiotics	0.05	0.05	0.05	0.05
Anticoccidials	0.01	0.01	0.01	0.01
Calculated composition, %				
Apparent metabolizable energy ^3^, kcal/kg	3150	3150	3200	3200
Dry matter ^2^	87.62	87.75	88.07	87.61
Crude protein ^2^	20.58	19.72	19.99	18.74
Ether extract ^2^	4.83	5.31	6.14	6.26
Crude ash ^2^	4.78	4.66	4.77	4.84
Calcium ^2^	0.84	0.78	0.82	0.86
Total phosphorus ^2^	0.53	0.52	0.57	0.55
Available phosphorus ^3^	0.36	0.36	0.36	0.35
Lysine ^3^	1.20	1.10	1.10	1.00
Methionine ^3^	0.60	0.55	0.57	0.50
Threonine ^3^	0.79	0.73	0.75	0.68
Tryptophan ^3^	0.23	0.22	0.23	0.23

^1^ Vitamin and mineral premix provided following nutrients per kg of diet: vitamin A, 12,000 IU; vitamin D_3_, 3000 IU; vitamin E, 15 mg; vitamin K, 2 mg; thiamine, 2 mg; riboflavin, 6 mg; pyridoxamine, 2 mg; vitamin B_12_, 0.03 mg; niacin, 45 mg; biotin, 0.15 mg; pantothenic acid, 15 mg folacin, 1 mg; Fe, 0.30 mg; Mn, 60 mg; Zn, 40 mg; I, 0.90 mg; Se, 0.2 mg; Cu, 66 mg; Co 0.20 mg; ^2^ Analyzed values; ^3^ Calculated values.

**Table 2:** Effect of low-protein diet and exogenous protease on growth performance in broiler chickens.

Items	CP		ENZ		SEM	*p*-Value		
	LP	NP	−	+		CP	ENZ	INT
BW, g/bird								
8 days	163.4	163.4	163.4	163.4	0.711	0.944	0.968	0.969
21 days	962.0	973.0	951.6 ^b^	983.5 ^a^	10.011	0.332	0.007	0.182
35 days	2064	2095	2093	2066	46.615	0.532	0.584	0.318
BWG, g/day/bird								
8 days to 21 days	57.98	58.77	57.48 ^b^	59.27 ^a^	0.567	0.210	0.006	0.133
22 days to 35 days	97.52	99.50	99.77	97.25	1.396	0.168	0.079	0.635
8 days to 35 days	77.42	79.14	77.93	78.62	0.887	0.081	0.478	0.649
FI, g/day/bird								
8 days to 21 days	80.99	80.09	80.80	80.29	0.616	0.185	0.454	0.802
22 days to 35 days	153.8	153.9	154.1	153.6	1.734	0.945	0.797	0.524
8 days to 35 days	117.4	116.3	116.7	117.0	0.971	0.290	0.806	0.927
FCR, g/day/bird								
8 days to 21 days	1.399 ^a^	1.364 ^b^	1.407 ^a^	1.355 ^b^	0.012	0.009	<.001	0.117
22 days to 35 days	1.580	1.548	1.546 ^b^	1.582 ^a^	0.016	0.062	0.034	0.927
8 days to 35 days	1.517 ^a^	1.484 ^b^	1.498	1.503	0.011	<0.001	0.405	0.395

^a,b^ Values (*n* = 12/treatment) having a different superscript with a row differ significantly (*p* < 0.05). CP = crude protein; NP = normal protein diet; LP = low-protein diet; ENZ = protease; INT = interaction; BW = body weight; BWG = body weight gain; FI = feed intake; FCR = feed conversion ratio; SEM = standard errors of the means.

**Table 3:** Effect of low-protein diet and exogenous protease on relative organ weight (g/100 g of live body weight) in broiler chickens.

Item	CP	NP		LP		SEM	*p*-Value		
	ENZ	−	+	−	+		CP	ENZ	INT
Spleen									
Days 21		0.093	0.093	0.095	0.094	0.005	0.796	0.938	0.878
Days 35		0.107	0.109	0.106	0.106	0.007	0.736	0.934	0.909
Liver									
Days 21		3.180	3.539	3.329	3.643	0.129	0.333	0.013	0.861
Days 35		2.749	2.894	2.746	2.775	0.111	0.586	0.434	0.603
Pancreas									
Days 21		0.384	0.356	0.380	0.374	0.014	0.634	0.226	0.445
Days 35		0.242	0.266	0.258	0.236	0.008	0.420	0.906	0.010

CP = crude protein diet; NP = normal protein diet; LP = low-protein diet; ENZ = protease; INT = interaction. SEM = standard errors of the means.

**Table 4:** Effects of low-protein diet and exogenous protease on leg meat yield (g/100 g of live body weight) and meat quality in broiler chickens.

Item	CP	NP		LP		SEM	*p*-Value		
	ENZ	−	+	−	+		CP	ENZ	INT
Leg meat yield									
Days 21		6.975 ^b^	7.003 ^b^	7.317 ^a^	6.852 ^b^	0.107	0.377	0.047	0.026
Days 35		6.975	6.874	7.188	6.966	0.130	0.250	0.223	0.645
Cooking loss, %									
Days 21		27.95	26.13	27.21	25.69	1.340	0.662	0.220	0.911
Days 35		23.35	21.87	20.94	23.27	1.999	0.804	0.831	0.346
CIE L*									
Days 21		51.18	54.66	54.20	50.60	1.578	0.741	0.968	0.030
Days 35		55.64	54.01	56.18	52.59	0.949	0.642	0.009	0.307
CIE a*									
Days 21		4.712	4.157	4.085	4.668	0.478	0.904	0.977	0.240
Days 35		4.648	5.177	4.349	5.334	0.438	0.873	0.091	0.605
CIE b*									
Days 21		16.53	12.74	15.11	12.60	0.973	0.426	0.002	0.518
Days 35		14.51	13.78	14.30	13.41	0.548	0.594	0.148	0.879
pH									
Days 21		5.940	6.006	5.908	5.988	0.050	0.619	0.152	0.881
Days 35		6.236	6.225	6.295	6.303	0.056	0.225	0.979	0.862

^a,b^ Values (*n* = 12/treatment) having a different superscript with a row differ significantly (*p* < 0.05). CP = crude protein diet; NP = normal protein diet, LP = low-protein diet; ENZ = protease; INT = interaction; DM = dry matter; SEM = standard errors of the means; CIE L*a*b* = Commission Internationale de l’Eclairage color values: lightness (L*), redness (a*), and yellowness (b*).

**Table 5:** Effects of low-protein diet and exogenous protease on breast meat yield (g/100 g of live body weight) and meat quality in broiler chickens.

Item	CP	NP		LP		SEM	*p*-Value		
	ENZ	−	+	−	+		CP	ENZ	INT
Meat yield									
Days 21		7.317	7.451	7.174	7.333	0.173	0.453	0.401	0.940
Days 35		9.429	9.118	9.151	9.139	0.289	0.659	0.578	0.608
Cooking loss, %									
Days 21		20.96	24.16	24.71	23.35	1.075	0.178	0.396	0.040
Days 35		33.41	34.85	34.04	36.34	3.392	0.756	0.583	0.900
CIE L*									
Days 21		53.50 ^b^	54.60 ^b^	56.92 ^a^	54.76 ^b^	0.682	0.012	0.444	0.021
Days 35		53.40	51.90	53.68	53.84	1.142	0.336	0.562	0.470
CIE a*									
Days 21		2.271	1.721	1.857	1.368	0.340	0.265	0.134	0.929
Days 35		1.216	1.625	0.612	0.748	0.414	0.081	0.514	0.743
CIE b*									
Days 21		14.98	15.59	15.77	13.87	0.621	0.462	0.305	0.049
Days 35		14.42	13.81	13.58	13.65	0.387	0.204	0.493	0.380
pH									
Days 21		5.564 ^a^	5.546 ^a^	5.430 ^b^	5.520 ^ab^	0.033	0.018	0.276	0.106
Days 35		5.718	5.642	5.738	5.606	0.042	0.843	0.017	0.515

^a,b^ Values (*n* = 12/treatment) having a different superscript with a row differ significantly (*p* < 0.05). CP = crude protein; NP = normal protein diet; LP = low-protein diet; ENZ = protease; INT = interaction; DM = dry matter; SEM = standard errors of the means; CIE L*a*b* = Commission Internationale de l’Eclairage color values: lightness (L*), redness (a*), and yellowness (b*).

**Table 6:** Effect of low-protein diets and exogenous protease on tibia characteristics in broiler chickens.

Item	CP		ENZ		SEM	*p*-Value		
	LP	NP	−	+		CP	ENZ	INT
Weight, g/100 BW								
Days 21	0.847	0.851	0.842	0.856	0.017	0.848	0.404	0.880
Days 35	0.829	0.810	0.839	0.800	0.020	0.354	0.061	0.358
Width, cm								
Days 21	0.625	0.630	0.621	0.634	0.019	0.828	0.514	0.514
Days 35	0.813	0.796	0.796	0.813	0.022	0.461	0.461	0.271
Length, cm								
Days 21	7.650	7.730	7.721	7.659	0.070	0.264	0.376	0.859
Days 35	10.48	10.44	10.48	10.44	0.068	0.626	0.543	0.396
Strength, kgf								
Days 21	18.17	17.52	17.18	18.51	0.810	0.431	0.106	0.756
Days 35	42.90	41.17	42.23	41.85	1.791	0.340	0.832	0.199

CP = crude protein; NP = Normal protein diet; LP = low-protein diet; ENZ = protease; INT = interaction; BW = body weight; SEM = standard errors of the means.

**Table 7:** Effect of low-protein diet and exogenous protease on ileal morphology, secretory immunoglobulin A (sIgA), and *C. perfringens* counts in broiler chickens.

Item	CP		ENZ		SEM	*p*-Value		
	LP	NP	−	+		CP	ENZ	INT
Days 21								
Villus height, μm	719.6	642.7	717.5	644.8	44.182	0.110	0.130	0.841
Crypt depth, μm	184.3	165.4	183.2	166.5	14.093	0.215	0.272	0.789
VH:CD ratio	4.048	3.956	4.036	3.968	0.235	0.717	0.788	0.750
sIgA, μg/cm of ileal segment								
Days 21	4.787	4.319	4.966	4.141	0.721	0.534	0.275	0.783
Days 35	7.076	7.028	7.402	6.702	0.651	0.941	0.294	0.084
*C. perfringens*, log_10_ cfu/g								
Days 35	6.931	6.854	6.803	6.982	0.106	0.506	0.136	0.734

CP = crude protein; NP = normal protein diet; LP-low-protein diet; ENZ = protease; INT = interaction; VH: CD ratio = villus height to crypt depth ratio; SEM = standard errors of the means.

**Table 8:** Effects of low-protein diet and exogenous protease on concentrations (mM/g digesta) of cecal short-chain fatty acid (SCFA) in broiler chickens.

Item	CP	NP		LP		SEM	*p*-Value		
	ENZ	−	+	−	+		CPS	ENZ	INT
Days 21									
Acetate		14.89	20.02	22.42	16.20	2.954	0.542	0.857	0.067
Propionate		1.593	2.335	2.163	2.026	0.335	0.705	0.383	0.208
Isobutyrate		0.081	0.094	0.092	0.085	0.010	0.920	0.779	0.385
Butyrate		2.897	6.879	5.416	4.090	1.253	0.919	0.320	0.051
Isovalerate		0.201	0.255	0.272	0.240	0.036	0.472	0.768	0.279
Valerate		0.285	0.524	0.422	0.340	0.080	0.792	0.371	0.072
Lactate		0.419	0.522	0.529	0.502	0.056	0.494	0.566	0.325
BCFA		0.571	0.890	0.786	0.652	0.120	0.934	0.506	0.109
Total SCFA		19.51	30.41	31.01	23.09	4.400	0.644	0.742	0.043
Days 35									
Acetate		64.55	49.47	51.20	50.81	6.085	0.329	0.211	0.234
Propionate		5.852	5.111	5.046	5.362	0.429	0.521	0.622	0.224
Isobutyrate		0.229	0.216	0.226	0.223	0.023	0.925	0.727	0.828
Butyrate		19.43	14.01	14.09	17.42	3.167	0.762	0.743	0.174
Isovalerate		0.731	0.633	0.734	0.730	0.088	0.571	0.560	0.594
Valerate		1.199	0.922	1.041	1.066	0.114	0.952	0.278	0.195
Lactate		1.354	1.743	1.301	1.177	0.213	0.181	0.561	0.265
BCFA		2.145	1.770	2.001	2.019	0.175	0.769	0.319	0.275
Total SCFA		81.66	84.62	77.42	78.09	7.962	0.507	0.823	0.888

CP = crude protein; NP = normal protein diet; LP = low-protein diet; ENZ = protease; INT = interaction; BCFA = branched-chain fatty acid (isobutyrate + valerate + isovalerate); Total SCFA = total short-chain fatty acid (acetate + propionate + butyrate + isobutyrate + valerate + isovalerate); SEM = standard errors of the means.

**Table 9:** Effect of low-protein diet and exogenous protease on serum biological parameters in broiler chickens.

Item	CP		ENZ		SEM	*p*-Value		
	LP	NP	−	+		CP	ENZ	INT
Days 35								
TCHO, mg/dL	123.3	113.8	120.4	116.7	5.245	S	0.478	0.670
TG, mg/dL	84.38	71.42	75.04	80.75	9.470	0.178	0.550	0.160
HDL, mg/dL	70.67	66.59	67.96	69.30	4.180	0.334	0.751	0.209
TP, g/dL	3.300	3.179	3.254	3.225	0.120	0.317	0.808	0.808
ALB, g/dL	1.229	1.154	1.179	1.204	0.059	0.213	0.676	0.780
GLB, g/dL	2.071	2.025	2.075	2.021	0.067	0.500	0.426	0.854
ALB:GLB	0.592	0.570	0.566	0.596	0.018	0.231	0.119	0.810
GOT, IU/L	284.7	264.4	291.3	257.8	22.984	0.382	0.152	0.298
GPT, IU/L	4.167	4.000	4.125	4.042	0.299	0.580	0.782	0.782
UA, mg/dL	6.550 ^a^	4.975 ^b^	5.138	6.388	0.632	0.017	0.054	0.582
Creatinine, mg/dL	0.480 ^a^	0.377 ^b^	0.396	0.461	0.045	0.028	0.156	0.817
NO, μM	11.12	12.25	11.20	12.17	2.320	0.638	0.685	0.372

^a,b^ Values (*n* = 12/treatment) having a different superscript with a row differ significantly (*p* < 0.05). CP = crude protein; NP = normal protein diet; LP = low-protein diet; ENZ = protease; INT = interaction; TCHO = total cholesterol; TG = triglyceride; HDL = High density lipoprotein; TP = total protein; ALB = albumin; GLB = globulin; ALB: GLB = albumin to globulin ratio; GOT = glutamic oxalacetic transaminase; GPT = glutamic pyruvic transaminase; UA = uric acid; NO = nitric oxide; SEM = standard errors of the means.

## References

[1] National Research Council (1994). Nutrient Requirements of Poultry.

[2] Nir I., Nitsan Z., Mahagna M. (1993). Comparative growth and development of the digestive organs and of some enzymes in broiler and egg type chicks after hatching. Br. Poult. Sci..

[3] Le Heurou-Luron I., Lhoste E., Wicker-Planquarl C., Dakka N., Toullec R., Corring T., Guilloteau P., Puigserver A. (1993). Molecular aspects of enzyme synthesis in the exocrine pancreas with emphasis on development and nutritional regulation. Proc. Nutr. Soc..

[4] Wang X., Parsons C.M. (1998). Effect of raw material source, processing systems, and processing temperatures on amino acid digestibility of meat and bone meals. Poult. Sci..

[5] Lemme A., Ravindran V., Bryden W.L. (2004). Ileal digestibility of amino acids in feed ingredients for broilers. Worlds Poult. Sci. J..

[6] Applegate T.J., Angel R. (2014). Nutrient requirements of poultry publications: History and need for an update. J. Appl. Poult. Sci..

[7] Angel C.R., Saylor W., Vieira S.L., Ward N. (2011). Effects of a monocomponent protease on performance and protein utilization in 7- to 22-day-old broiler chickens. Poult. Sci..

[8] Cowieson A.J., Roos F.F. (2016). Toward optimal value creation through the application of exogenous mono-component protease in the diets of non-ruminants. Anim. Feed Sci. Technol..

[9] Kocher A., Choct M., Porter M.D., Broz J. (2002). Effects of feed enzymes on nutritive value of soyabean meal fed to broilers. Br. Poult. Sci..

[10] Huo G.C., Fowler V.R., Inborr J., Bedford M.R. The use of enzymes to denature antinutritive factors in soybean. Proceedings of the 2nd Workshop on ‘antinutritional factors (ANFs) in legume seed’.

[11] Kaczmarek S.A., Rogiewicz A., Mogielnicka M., Rutkowski A., Jones R.O., Slominski B.A. (2014). The effect of protease, amylase, and nonstarch polysaccharide-degrading enzyme supplementation on nutrient utilization and growth performance of broiler chickens fed corn-soybean meal-based diets. Poult. Sci..

[12] Mahmood T., Mirza M.A., Nawaz H., Shahid M., Athar M., Hussain M. (2017). Effect of supplementing exogenous protease in low protein poultry by-product meal based diets on growth performance and nutrient digestibility in broilers. Anim. Feed Sci. Technol..

[13] Marsman G.J., Gruppen H., Van der Poel A.F., Kwakkel R.P., Verstegen M.W., Voragen A.G. (1997). The effect of thermal processing and enzyme treatments of soybean meal on growth performance, ileal nutrient digestibilities, and chyme characteristics in broiler chicks. Poult. Sci..

[14] Cowieson A.J., Zaefarian F., Knap I., Ravindran V. (2017). Interactive effects of dietary protein concentration, a mono-component exogenous protease and ascorbic acid on broiler performance, nutritional status and gut health. Anim. Prod. Sci..

[15] Kim D.H., Han S.M., Keum M.C., Lee S., An B.K., Lee S.-R., Lee K.-W. (2018). Evaluation of bee venom as a novel feed additive in fast-growing broilers. Br. Poult. Sci..

[16] Joo H.-S., Kumar C.G., Park G.-C., Paik S.R., Chang C.-S. (2003). Oxidant and SDS-stable alkaline protease from *Bacillus calusii* I-52: Production and some properties. J. Appl. Microbiol..

[17] Kim H.J., Cho J.H., Chen Y.J., Yoo J.S., Min B.J., Kim I.H. (2007). Effects of coastal mud-flat bacteria origin protease supplementation on laying performance, nutrient digestibility and total protein concentration of serum in laying hens. Korean J. Poult. Sci..

[18] Kim H.J., Cho J.H., Chen Y.J., Yoo J.S., Min B.J., Jang J.S., Kang K.R., Kim I.H. (2007). Effects of mud flat bacteria origin protease supplementation by crude protein level on growth performance, nutrient digestibility, total protein and BUN concentration in broiler. Korean J. Poult. Sci..

[19] Kim H.W., Yan F.F., Hu J.Y., Cheng H.W., Kim Y.H. (2016). Effects of probiotics feeding on meat quality of chicken breast during postmortem storage. Poult. Sci..

[20] Lee S., Kim D.-H., Keum M.-C., Han E., An B.-K., Chang H.-H., Choi Y.-H., Moon B.-H., Lee K.W. (2018). Effects of fumonisin B1 and mycotoxin binders on growth performance, tibia characteristics, gut physiology, and stress indicators in broiler chickens raised in different stocking densities. Poult. Sci..

[21] Aftab U., Ashraf M., Jiang Z. (2006). Low protein diets for broilers. World Poult. Sci. J..

[22] Yang H., Yang Z., Wang Z., Wang W., Huang K., Fan W., Jia T. (2015). Effects of early dietary energy and protein dilution on growth performance, nutrient utilization and internal organs of broilers. Ital. J. Anim. Sci..

[23] Ghazi S., Rooke J.A., Galbraith H. (2003). Improvement of the nutritive value of soybean meal by protease and a-galactosidase treatment in broiler cockerels and broiler chicks. Br. Poult. Sci..

[24] Law F.L., Zulkifli I., Soleimani A.F., Liang J.B., Awad E.A. (2018). The effects of low-protein diets and protease supplementation on broiler chickens in a hot and humid tropical environment. Asian-Australas. J. Anim. Sci..

[25] Walk C.L., Cowieson A.J., Remus J.C., Novak C.L., McElroy A.P. (2011). Effects of dietary enzymes on performance and intestinal goblet cell number of broilers exposed to a live coccidia oocyst vaccine. Poult. Sci..

[26] Ding X.M., Li D.D., Li Z.R., Wang J.P., Zeng Q.F., Bai S.P., Su Z.W., Zhang K.Y. (2016). Effects of dietary crude protein levels and exogenous protease on performance, nutrient digestibility, trypsin activity and intestinal morphology in broilers. Livest. Sci..

[27] Kamel N.F., Ragaa M., El-Banna R.A., Mohamed F.F. (2015). Effects of a monocomponent protease on performance parameters and protein digestibility in broiler chickens. Agric. Agric. Sci. Procedia..

[28] Cowieson A.J., Lu H., Ajuwon K.M., Knap I., Adeola O. (2016). Interactive effects of dietary protein source and exogenous protease on growth performance, immune competence and jejunum health of broiler chickens. Anim. Prod. Sci..

[29] Xu X., Wang H.L., Pan L., Ma X.K., Tian Q.Y., Xu Y.T., Long S.F., Zhang Z.H., Piao X.S. (2017). Effects of coated proteases on the performance, nutrient retention, gut morphology and carcass traits of broilers fed corn or sorghum based diets supplemented with soybean meal. Anim. Feed Sci. Technol..

[30] Stefanello C., Vieira S.L., Rios H.V., Simões C.T., Sorbara J.O.B. (2016). Energy and nutrient utilisation of broilers fed soybean meal from two different Brazilian production areas with an exogenous protease. Anim. Feed Sci. Technol..

[31] Rehman Z.U., Kamran J., El-Hack M.A., Alagawany M., Bhatti S.A., Ahmad G., Saleem A., Ullah Z., Yameen R.M.K., Ding C. (2018). Influence of low-protein and low-amino acid diets with different sources of protease on performance, carcasses and nitrogen retention of broiler chickens. Anim. Prod. Sci..

[32] Erdaw M.M., Perez-Maldonado R.A., Iji P.A. (2018). Supplementation of broiler diets with high levels of microbial protease and phytase enables partial replacement of commercial soybean meal with raw, full-fat soybean. J. Anim. Physiol. Anim. Nutr..

[33] Mahmood T., Mirza M.A., Nawaz H., Shahid M. (2017). Effect of different exogenous proteases on growth performance, nutrient digestibility, and carcass response in broiler chickens fed poultry by-product meal-based diets. Livest. Sci..

[34] Mohammed A.A., Habib A.B., Eltrefi A.M., Shulukh E.S.A., Abubaker A.A. (2018). Effect of different levels of multi-enzymes (Natuzyme Plus^®^) on growth performance, carcass traits and meat quality of broiler chicken. Asian J. Anim. Vet. Adv..

[35] Brenes A., Smith M., Guenter W., Marquardt R.R. (1993). Effect of enzyme supplementation on the performance and digestive tract size of broiler chickens fed wheat- and barley-based diets. Poult. Sci..

[36] Gracia M.I., Araníbar M., Lázaro R., Medel P., Mateos G.G. (2003). α-Amylase supplementation of broiler diets based on corn. Poult. Sci..

[37] Wang Z.R., Qiao S.Y., Lu W.Q., Li D.F. (2005). Effects of enzyme supplementation on performance, nutrient digestibility, gastrointestinal morphology, and volatile fatty acid profiles in the hindgut of broilers fed wheat-based diets. Poult. Sci..

[38] Onderci M., Sahin N., Sahin K., Cikim G., Aydin A., Ozercan I., Aydin S. (2006). Efficacy of supplementation of α-amylase-producing bacterial culture on the performance, nutrient use, and gut morphology of broiler chickens fed a corn-based diet. Poult. Sci..

[39] Yuan J., Yao J., Yang F., Yang X., Wan X., Han J., Wang Y., Chen X., Liu Y., Zhou Z., Zhou N., Feng X. (2008). Effects of supplementing different levels of a commercial enzyme complex on performance, nutrient availability, enzyme activity and gut morphology of broilers. Asian-Australas. J. Anim. Sci..

[40] Saláková A., Straková E., Válková V., Buchtová H., Steinhauserová I. (2009). Quality indicators of chicken broiler raw and cooked meat depending on their sex. Acta Vet. Brno..

[41] Mir N.A., Rafiq A., Kumar F., Singh V., Shukla V. (2017). Determinations of broiler chicken meat quality and factors affecting them: A review. J. Food Sci. Technol..

[42] Choct M. (2006). Enzymes for the feed industry: Past, present and future. Worlds Poult. Sci. J..

[43] Yason C.V., Summers B.A., Schat K.A. (1987). Pathogenesis of rotavirus infection in various age groups of chickens and turkeys: Pathology. Am. J. Vet. Res..

[44] Nabizadeh A., Golian A., Hassanabadi A., Zerehdaran S. (2017). Effects of nutrient density and exogenous enzymes in starter diet on performance, intestinal microflora, gut morphology and immune response of broiler chickens. Rev. Bras. Cienc. Avic..

[45] Mantis N.J., Rol N., Corthésy B. (2011). Secretory IgA’s complex roles in immunity and mucosal homeostasis in the gut. Mucosal Immunol..

[46] Yang X.J., Li W.L., Feng Y., Yao J.H. (2011). Effects of immune stress on growth performance, immunity, and cecal microflora in chickens. Poult. Sci..

[47] Peng Q., Zeng X.F., Zhu J.L., Wang S., Liu X.T., Hou C.L., Thacker P.A., Qiao S.Y. (2016). Effects of dietary Lactobacillus plantarum B1 on growth performance, intestinal microbiota, and short chain fatty acid profiles in broiler chickens. Poult. Sci..

[48] Bjerrum L., Engberg R.M., Leser T.D., Jensen B.B., Finster K., Pedersen K. (2006). Microbial community composition of the ileum and cecum of broiler chickens as revealed by molecular and cellular-based techniques. Poult. Sci..

[49] Topping D.L., Clifton P.M. (2001). Short-chain fatty acids and human colonic function: Roles of resistant starch and nonstarch polysaccharides. Physiol. Rev..

[50] Qaisrani S.N., Moquet P.C.A., Van Krimpen M.M., Kwakkel R.P., Verstegen M.W.A., Hendriks W.H. (2014). Protein source and dietary structure influence growth performance, gut morphology, and hindgut fermentation characteristics in broilers. Poult. Sci..

[51] Yan F., Dibner J.J., Knight C.D., Vazquez-Anon M. (2016). Effect of carbohydrase and protease on growth performance and gut health of young broilers fed diets containing rye, wheat, and feather meal. Poult. Sci..

[52] Schmidt E.M.S., Locatelli-Dittrich R., Santin E., Paulillo A.C. (2007). Patologia clínica em aves de produção–uma ferramenta para monitorar a sanidade avícola–revisão. Farriers Mag..

[53] Corzo A., Fritts C.A., Kidd M.T., Kerr B.J. (2005). Response of broiler chicks to essential and non-essential amino acid supplementation of low crude protein diets. Anim. Feed Sci. Technol..

[54] Swennen Q., Janssens G.J., Collin A., Le Bihan-Duval E., Verbeke K., Decuypere E., Buyse J. (2006). Diet-induced thermogenesis and glucose oxidation in broiler chickens: influence of genotype and diet composition. Poult. Sci..

[55] Namroud N.F., Shivazad M., Zaghari M. (2008). Effects of fortifying low crude protein diet with crystalline amino acids on performance, blood ammonia level, and excreta characteristics of broiler chicks. Poult. Sci..

[56] Hernández F., López M., Martínez S., Megías M.D., Catalá P., Madrid J. (2012). Effect of low-protein diets and single sex on production performance, plasma metabolites, digestibility, and nitrogen excretion in 1-to 48-day-old broilers. Poult. Sci..

[57] Donsbough A.L., Powell S., Waguespack A., Bidner T.D., Southern L.L. (2010). Uric acid, urea, and ammonia concentrations in serum and uric acid concentrations in excreta as indicators of amino acid utilization in diets for broilers. Poult. Sci..

[58] Arczewska-Włosek A., Świątkiewicz S., Ognik K., Józefiak D. (2018). Effect of dietary crude protein level and supplemental herbal extract blend on selected blood variables in broiler chickens vaccinated against coccidiosis. Animals.

